# Enhancement of Hypoxia-Induced Autophagy via the HIF-1apha/BNIP3 Pathway Promotes Proliferation and Myogenic Differentiation of Aged Skeletal Muscle Satellite Cells

**DOI:** 10.3390/life16010144

**Published:** 2026-01-16

**Authors:** Li Zhou, Chenghao Feng, Jinrun Lin, Minghao Geng, Danni Qu, Jihao Xing, Hao Lin, Xiaoqi Ma, Ryosuke Nakanishi, Noriaki Maeshige, Hiroyo Kondo, Hidemi Fujino

**Affiliations:** Department of Rehabilitation Science, Kobe University Graduate School of Health Sciences, 7-10-2, Tomogaoka, Suma-ku, Kobe 654-0142, Japan; zhouli940125@163.com (L.Z.); fengchenghao_0813@yahoo.co.jp (C.F.); ljr935616@126.com (J.L.); mickey.geng@outlook.com (M.G.); qdn960828@gmail.com (D.Q.); xingjihao1995@gmail.com (J.X.); linhao_china1995@yahoo.co.jp (H.L.); mxq213k212k@gmail.com (X.M.); nakanishi.kobe@gmail.com (R.N.); nmaeshige@pearl.kobe-u.ac.jp (N.M.); kondohiroyo@gmail.com (H.K.)

**Keywords:** hypoxia, aging satellite cells, autophagy

## Abstract

Aged skeletal muscle satellite cells (MuSCs) exhibit impaired autophagy-related activity, reduced proliferative capacity, and compromised myogenic differentiation, which collectively contribute to defective muscle regeneration during aging. However, whether hypoxia-driven modulation of autophagy-related activity can improve aged MuSC function and the underlying molecular mechanisms remain incompletely understood. In this study, aged MuSCs were divided into three groups: normoxia, hypoxia, and hypoxia combined with an autophagy inhibitor. Aged MuSCs exhibited a decreased LC3B-II/LC3B-I ratio and Beclin-1 expression, together with elevated p62 levels, indicating altered autophagy-related activity. Hypoxic culture was associated with enhanced autophagy-related activity in aged MuSCs, accompanied by HIF-1α stabilization, BNIP3 upregulation, and reduced p62 accumulation. Functionally, hypoxia significantly promoted the proliferation and myogenic differentiation of aged MuSCs. Pharmacological inhibition of autophagy using 3-methyladenine, as well as BNIP3 suppression, markedly attenuated these hypoxia-induced functional improvements. Collectively, these findings suggest that hypoxia is associated with improved proliferative and myogenic capacities of aged MuSCs, potentially involving autophagy-related activity regulated by the HIF-1α/BNIP3 pathway. This study provides insight into the relationship between hypoxic signaling and autophagy in aged MuSCs and may inform future strategies aimed at improving muscle regeneration during aging.

## 1. Introduction

Age-related sarcopenia is associated with a defective regenerative ability caused by a reduced in the number and function of resident adult muscle stem cells (satellite cells) [1]. As muscle stem cells with self-renewal capacity, satellite cells activate in response to tissue damage and differentiate into muscle cells to reconstruct muscle fibers [2]. However, aging leads to a reduction in satellite cell numbers and functional impairment, resulting in an inability to maintain muscle regeneration capacity [3].

Multiple lines of evidence link the age-related loss of satellite-cell regenerative capacity to disrupted autophagy [4,5,6]. Aguilera et al.’s study in aged muscle stem cells indicates that failure of basal autophagy precipitates mitochondrial dysfunction and oxidative stress, activates senescence programs, and that restoring autophagic activity rejuvenates these cells and rescues regeneration [7]. According to Galasso’s research, aging depresses autophagic—particularly mitophagic—flux, leading to inadequate clearance of damaged organelles, accumulation of reactive oxygen species, and blunted myogenesis in vivo and in vitro [8]. Taken together, these reports support the view that impaired autophagic flux is a proximate driver of regenerative failure in aged satellite cells, and that strategies aimed at re-establishing autophagy constitute a rational route to reactivate stem-cell function and improve muscle repair [9,10,11].

Starvation, exercise, and rapamycin are established inducers of autophagy [12,13,14]. Nonetheless, in the context of age-related conditions, rapamycin confers only modest functional improvements [15], whereas exercise and fasting often exhibit poor long-term adherence and yield limited regenerative benefit in older adults with frailty or multimorbidity [16,17]. Hypoxia, a physiologically relevant microenvironmental cue in muscle tissue, has emerged as a potent regulator of autophagy [18]. The hypoxia-inducible factor-1α (HIF-1α) pathway mediates cellular adaptation to low oxygen levels, and its downstream effector BNIP3 is known to promote mitophagy [19]. Although hypoxia-induced autophagy via the HIF-1α/BNIP3 signaling axis has been implicated in other stem cell systems [20,21], its role in aged skeletal muscle satellite cells remains poorly understood.

This study evaluates how hypoxia-induced autophagy affects the proliferation and myogenic differentiation of aged skeletal muscle satellite cells. We hypothesize that hypoxia-driven autophagy enhances both processes, thereby providing a rationale for new therapeutic strategies for sarcopenia.

## 2. Materials and Methods

### 2.1. Cell Culture

Skeletal muscle satellite cells were purchased from the RIKEN BioResource Research Center (BRC) Cell Bank (RCB2366, Tsukuba, Japan) and maintained under standard culture conditions according to the manufacturer’s protocol. Cells were seeded in 60 mm culture dishes and, upon reaching approximately 80% confluence, were allocated to three groups: normoxia, hypoxia, and hypoxia + 3-MA.

For the interventions, the normoxia group was maintained under normoxic conditions (21% O_2_ and 5% CO_2_), whereas the hypoxia and hypoxia + 3-MA groups were cultured in a tri-gas (O_2_/N_2_/CO_2_) hypoxia incubator (custom-made) set to 3% O_2_ and 5% CO_2_ (balanced with N_2_). After 24 h under the indicated conditions, autophagy, cell proliferation, and myogenic differentiation were assessed. All experiments were performed in triplicate and independently repeated three times with similar results.

### 2.2. Cellular Senescence Model

Based on Khor et al.’s report, we established a high-passage satellite cell model [22]. Briefly, cells were plated in 100 mm culture dishes and maintained at 37 °C in a humidified incubator under normoxia (21% O_2_) with 5% CO_2_. The culture medium was Dulbecco’s Modified Eagle Medium (DMEM) supplemented with 20% fetal bovine serum (FBS) and 1% penicillin–streptomycin (P/S). Cells were passaged at approximately 80% confluence and serially subcultured until proliferation ceased, after which they were cryopreserved at −80 °C.

### 2.3. Assessment of Cellular Senescence

For cellular senescence assays, satellite cells from the young and old groups were cultured separately in 35 mm culture dishes. Cellular senescence was assessed using a Senescence-Associated β-Galactosidase Staining Kit (#9860, Cell Signaling Technology, Danvers, MA, USA) according to the manufacturer’s instructions. At ~80% confluence, cells were washed once with 1× PBS, fixed with the kit fixative for 15 min at room temperature, washed twice, and incubated in the SA-β-gal staining solution. Dishes were sealed with Parafilm to limit evaporation and crystal formation and incubated overnight at 37 °C without CO_2_. Bright-field images were acquired at 200× using a BZ-X810 microscope (Keyence, Osaka, Japan) with identical imaging settings across groups. Three independent experiments were performed (n = 3). SA-β-Gal–positive (blue) cells were quantified by ImageJ-assisted manual counting (ImageJ, NIH). For each experiment, three non-overlapping fields were analyzed (one field from the center of the dish and two fields from peripheral regions in diagonal quadrants). Cells showing distinct cytoplasmic blue staining were classified as SA-β-Gal–positive. Normalization was performed by expressing SA-β-Gal positivity as the percentage of positive cells among total cells per field: (SA-β-Gal–positive cells/total cells) × 100, and values were averaged across the three fields for each experiment. To reduce potential bias, images were acquired using identical microscope settings across groups, and the same field-sampling approach and scoring criterion were applied consistently to all images.

### 2.4. Autophagy Inhibitor Treatment

To examine the role of autophagy in MuSCs proliferation and differentiation, the autophagy inhibitor 3-methyladenine (3-MA; 5 mM; HY-19312, MCE, Monmouth Junction, NJ, USA) was dissolved in complete growth medium, sterile-filtered through a 0.22 µm filter, and applied by replacing the culture medium in the hypoxia + 3-MA group for the indicated durations.

### 2.5. Western Blot

After the interventions, cells were lysed on ice in RIPA buffer supplemented with protease and phosphatase inhibitor cocktails (1% each, *v*/*v*) to extract total protein. Equal amounts of protein were resolved by 8–15% SDS–polyacrylamide gel electrophoresis (SDS–PAGE) and transferred to PVDF membranes. Membranes were blocked for 1 h at room temperature in 5% nonfat dry milk in TBST (Tris-buffered saline with 0.1% Tween-20) and incubated overnight at 4 °C with primary antibodies (1:1000 in TBST): LC3B (#12741, Cell Signaling Technology, Danvers, MA, USA), SQSTM1/p62 (#39749; Cell Signaling Technology, Danvers, MA, USA), and Beclin-1 (#3495; Cell Signaling Technology, Danvers, MA, USA). After three washes with TBST, membranes were incubated with the appropriate HRP-conjugated secondary antibodies for 2 h at room temperature. The proteins were detected using EzWestLumi One (ATTO). Finally, images were captured using the LuminoGraphI imaging system (ATTO). β-actin served as the loading control. Band intensities were quantified using ImageJ (version 1.53a; National Institutes of Health, Bethesda, MD, USA).

### 2.6. Immunofluorescence

After removing the culture medium, cells were fixed with 4% paraformaldehyde (sc-281692; Santa Cruz Biotechnology, Dallas, TX, USA) at room temperature for 30 min. Cells were then permeabilized with 0.1% Triton X-100 in PBS for 10 min at RT and blocked with EzBlock Chemi (ATTO) for 1 h. Primary antibodies, diluted in PBS containing 0.3% Triton X-100 at the indicated dilutions, were applied overnight at 4 °C: HIF-1α (diluted 1:400 in 0.3% Triton X-100; #36169; Cell Signaling Technology, Danvers, MA, USA), BNIP3 (diluted 1:800 in 0.3% Triton X-100; #44060; Cell Signaling Technology, Danvers, MA, USA), Ki-67 (diluted 1:400 in 0.3% Triton X-100; #9129S; Cell Signaling Technology, Danvers, MA, USA), MyoD (diluted 1:50 in 0.3% Triton X-100;sc-377460; Santa Cruz Biotechnology, Dallas, TX, USA), myogenin (diluted 1:50 in 0.3% Triton X-100;sc-12732; Santa Cruz Biotechnology, Dallas, TX, USA). The next day, cells were washed three times with 1× PBS, incubated with the appropriate fluorophore-conjugated secondary antibodies for 1 h at RT in the dark, and counterstained with DAPI for 5 min at 37 °C. Images were acquired on a fluorescence microscope, and fluorescence signals were quantified using ImageJ.

### 2.7. Cell Counting Kit-8

Cell proliferation was quantified using the Cell Counting Kit-8 assay (CCK-8; CK04, Dojindo, Kumamoto, Japan). MuSCs were seeded in 96-well plates (technical triplicates per condition). After the indicated treatments, the medium was replaced with 100 µL fresh growth medium containing 10 µL CCK-8 reagent per well (final 10% *v*/*v*) and incubated at 37 °C for 1 h protected from light. Absorbance at 450 nm was read on a microplate reader. Blank wells were used for background subtraction, and values were normalized to the Normal control.

### 2.8. Wound-Healing Assay

Cells were seeded in 6-well plates and grown to a confluent monolayer (~100% confluence). Uniform linear scratches were made with a 200 µL pipette tip under identical conditions across wells. Detached cells were removed by two PBS washes, and group-specific treatments were applied as indicated. Images were acquired at 0 h and 24 h using a fluorescence microscope. Wound areas were quantified in ImageJ using identical ROIs; wound closure (%) was calculated as (A_0_ − A_24_)/A_0_ × 100.

### 2.9. Myogenic Differentiation

Before myogenic differentiation, media in all groups were replaced with differentiation medium (DMEM supplemented with 2% horse serum and 1% penicillin–streptomycin). In the inhibitor group, 3-methyladenine (3-MA; 5 mM) was continued in the medium. Group-specific treatments were applied as indicated. Immunofluorescence staining was performed at marker-specific time points, and fluorescence intensity was quantified in ImageJ using identical acquisition settings with background subtraction.

### 2.10. Statistical Analysis

All experiments were performed with three or more independent biological replicates. Data are presented as mean ± standard error of the mean (SEM). Statistical analyses were conducted using GraphPad Prism 10.4.1 (GraphPad Software, La Jolla, CA, USA). Prior to parametric analyses, data normality was assessed using the Shapiro–Wilk test. Homogeneity of variances was evaluated using the F-test for two-group comparisons or the Brown–Forsythe test for multiple-group comparisons. No major deviations from these assumptions were detected. Group differences were analyzed using an unpaired two-tailed Student’s *t*-test or one-way analysis of variance (ANOVA), followed by Tukey’s post hoc multiple-comparison test. Statistical significance was defined as *p* < 0.05.

## 3. Results

### 3.1. Detection of Cellular Senescence by SA-β-Gal

**Figure 1:**
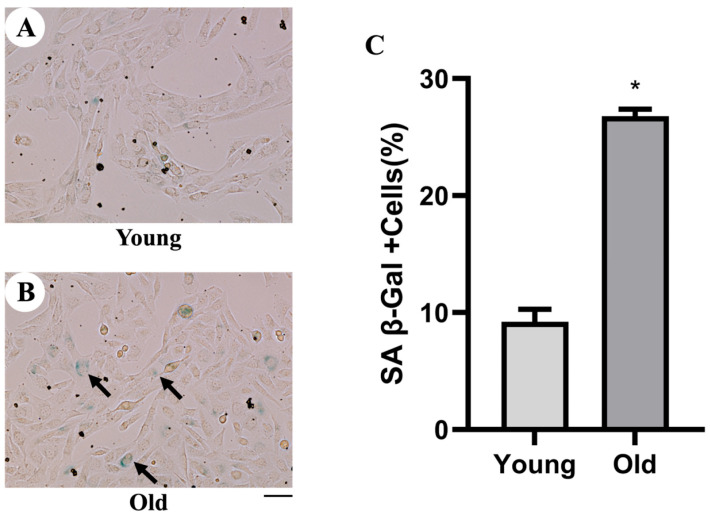
Detection of cellular senescence by Senescence-Associated β-Galactosidase assay. (**A**) Representative bright-field images of SA-β-gal staining in the young group. (**B**) Representative bright-field images of SA-β-gal staining in the old group. Arrows indicate SA-β-gal–positive cells (blue staining). (**C**) Analysis of the mean percentage of SA-β-Gal–positive cells. Scale bar = 50 μm. * Significant difference compared to young cells (*p* < 0.05). The data are expressed as the mean ± SEM (n = 3) of three experiments.

Cumulative aging was achieved by serial passaging in vitro. Specifically, MuSCs in the young group were used at passages < 3, whereas MuSCs in the old group were used at passages > 15, with the endpoint defined by a marked and sustained slowing of proliferation approaching growth arrest during continuous passaging. SA-β-gal staining was performed to evaluate senescence-associated staining in the young and old groups (Figure 1). The young group showed minimal blue staining with few SA-β-gal–positive cells (Figure 1A), whereas the old group exhibited more prominent cytoplasmic staining and a higher number of positive cells (Figure 1B). Accordingly, quantification revealed a significantly greater proportion of SA-β-gal–positive cells in the old group (Figure 1C). Collectively, these findings suggest increased senescence-associated staining in the old group and support the use of this serial-passaging model in subsequent experiments.

### 3.2. HIF-1α and BNIP3 Expression by Immunofluorescence

**Figure 2:**
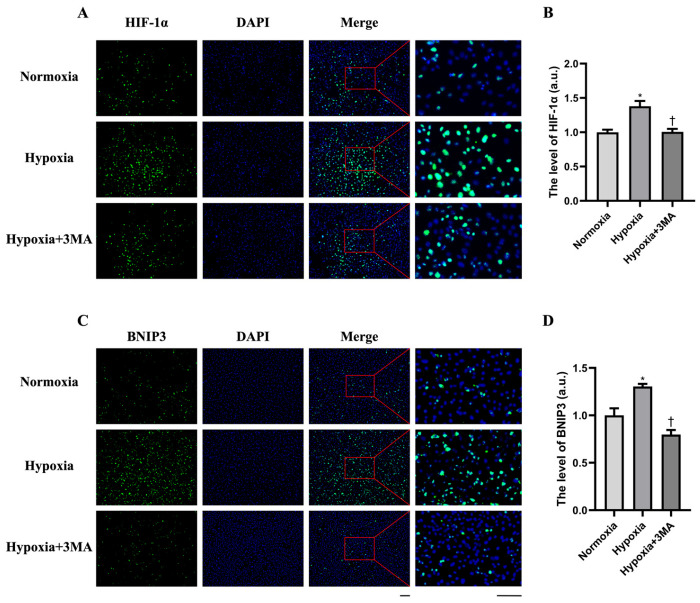
Detection of HIF-1α and BNIP3 expression by immunofluorescence staining. (**A**) Fluorescence quantification of HIF-1α. (**B**) Quantitative analysis of HIF-1α fluorescence. (**C**) Fluorescence quantification of BNIP3. (**D**) Quantitative analysis of BNIP3 fluorescence. DAPI staining is shown in blue, and both HIF-1α and BNIP3 are shown in green. Scale bar = 100 μm. The data are expressed as the mean ± SEM (n = 3) of three experiments. *,† indicate significant differences from Normoxia and Hypoxia, respectively; *p* < 0.05.

Given that HIF-1α is the principal transcription factor activated under hypoxia, we first assessed its expression across groups (Figure 2A). Quantitative analysis showed that HIF-1α protein levels were significantly higher in the Hypoxia groups than in the Normoxia and Hypoxia+3MA group, whereas no significant difference was observed between Normoxia and Hypoxia+3MA group (Figure 2B). This suggests indicating hypoxia-induced stabilization/activation of HIF-1α. To determine whether this activation affected a downstream target, BNIP3 was next analyzed (Figure 2C). Fluorescence quantification demonstrated that BNIP3 expression was significantly elevated in the Hypoxia group relative to both Normoxia and Hypoxia+3MA group, whereas no significant difference was detected between Normoxia and Hypoxia+3MA group (Figure 2D).

### 3.3. Autophagy-Related Proteins

**Figure 3:**
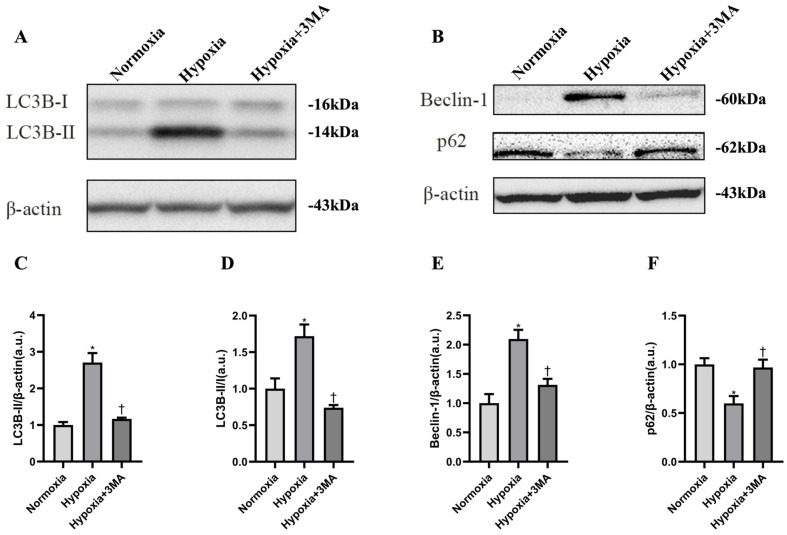
Detection of autophagy-related protein expression by WB. (**A**) WB results of LC3B. (**B**) WB results of Beclin-1 and p62. (**C**) Grayscale analysis of LC3B-II/β-actin. (**D**) Grayscale analysis of LC3B-II/I. (**E**) Grayscale analysis of Beclin-1/β-actin. (**F**) Grayscale analysis of p62/β-actin. The data are expressed as the mean ± SEM (n = 3) of three experiments. *,† indicate significant differences from Normoxia and Hypoxia, respectively (*p* < 0.05).

To determine whether HIF-1α and BNIP3 expression is associated with autophagic flux, the expression levels of the autophagy markers Beclin-1, LC3B, and p62 protein were analyzed by Western blotting (Figure 3A,B). Densitometric analysis showed that the LC3B-II/β-actin and LC3B-II/LC3B-I ratios, as well as Beclin-1 and p62, differed significantly in the Hypoxia group compared with both the Normoxia and Hypoxia+3MA groups, whereas no significant differences were observed between Normoxia and Hypoxia+3MA group (Figure 3C–F). These findings indicate that hypoxia successfully activates autophagy; however, in the presence of the autophagy inhibitor, 24 h of hypoxia is insufficient to overcome the inhibitory effect.

### 3.4. Cell Proliferation by Immunofluorescence and CCK-8

**Figure 4:**
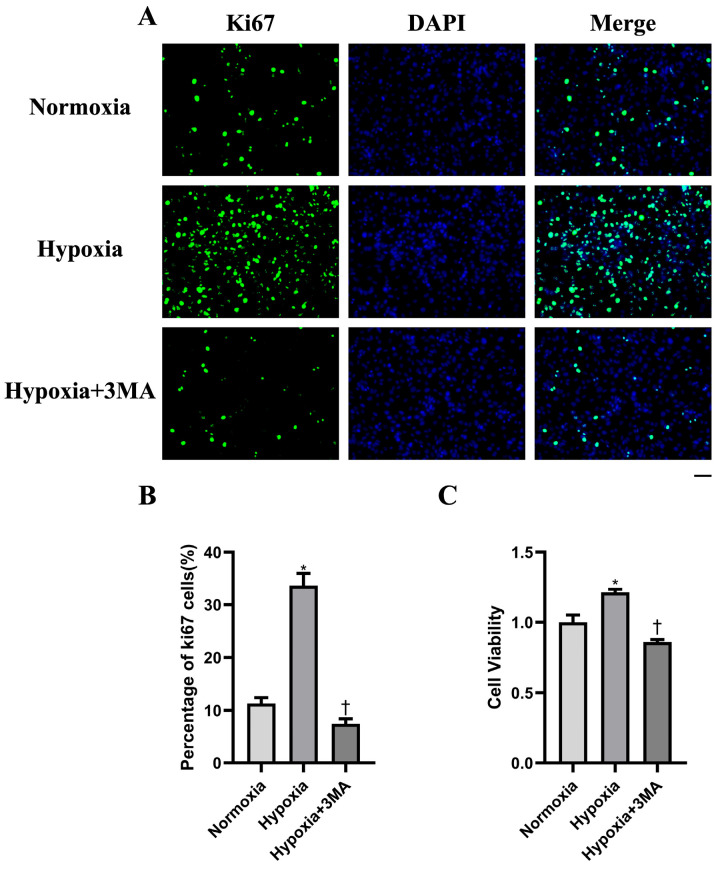
Assessment of cell proliferation using Ki67 immunofluorescence staining and the CCK-8 assay. (**A**) Immunofluorescence staining results of Ki67. (**B**) Quantification of Ki-67 positive cells (percentage). (**C**) Analysis of CCK-8 assay results. DAPI staining is shown in blue, Ki67 staining is shown in green. Scale bar = 100 μm. The data are expressed as the mean ± SEM (n = 3) of three experiments. *,† indicate significant differences from Normoxia and Hypoxia, respectively (*p* < 0.05).

To test whether autophagic activity is associated with cell proliferation, we assessed proliferation by Ki67 immunofluorescence and the CCK-8 assay (Figure 4A,C). Representative Ki67 immunofluorescence images are shown (Figure 4A). The percentage of Ki67-positive cells was significantly higher in the Hypoxia group than in Normoxia and Hypoxia+3MA group (Figure 4B). CCK-8 measurements mirrored the Ki67 findings (Figure 4C). Collectively, these data indicate that hypoxia-induced autophagy promotes the proliferation of aged skeletal muscle satellite cells.

### 3.5. Cell Migration by Scratch-Wound Assay

**Figure 5:**
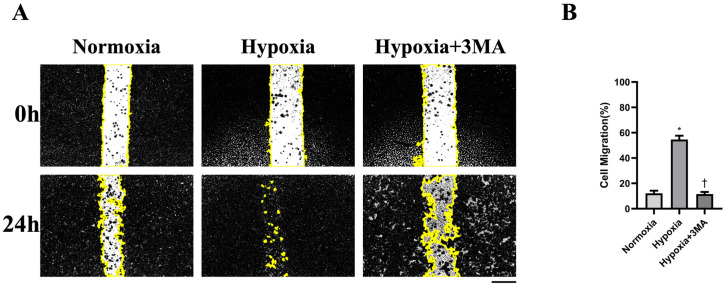
Detection of cell migration using the wound-healing assay. (**A**) Wound-healing assay. (**B**) Analysis of wound-healing assay results (percentage). The data are expressed as the mean ± SEM (n = 3) of three experiments. The yellow area indicates the wound (cell-free) region used for quantification of cell migration. Scale bar = 50 μm. *,† indicate significant differences from Normoxia and Hypoxia, respectively (*p* < 0.05).

Cell migratory capacity is shown by representative scratch-wound images (Figure 5A). The percentage of wound closure indicated that the Hypoxia group differed significantly from both the Normoxia and Hypoxia+3MA groups (Figure 5B).

### 3.6. Myogenic Differentiation by Immunofluorescence

**Figure 6:**
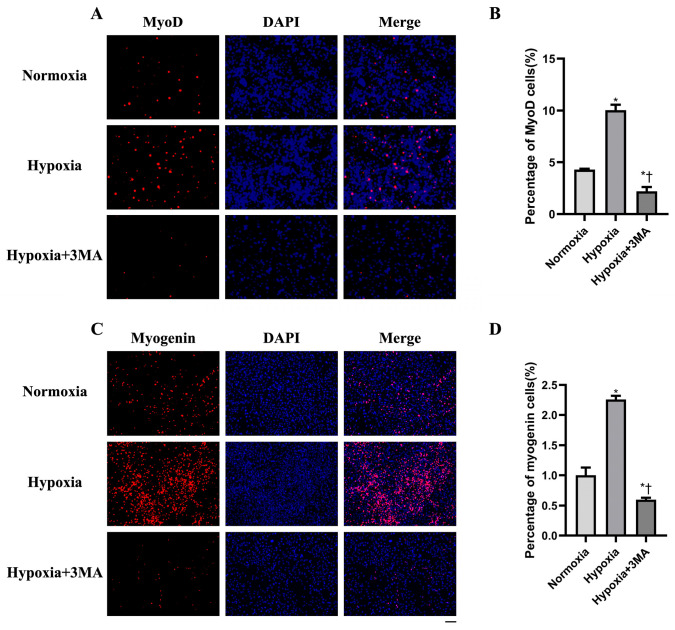
Detection of MyoD and myogenin expression by immunofluorescence staining. DAPI staining is shown in blue, and both MyoD and myogenin are shown in red. (**A**) Immunofluorescence staining results of MyoD. (**B**) Analysis of MyoD assay results. (**C**) Immunofluorescence staining results of myogenin. (**D**) Analysis of myogenin assay results. Scale bar = 100 μm. The data are expressed as the mean ± SEM (n = 3) of three experiments. *,† indicate significant differences from Normoxia and Hypoxia, respectively (*p* < 0.05).

Immunofluorescence staining was used to assess expression of the myogenic regulators MyoD and myogenin (Figure 6). Following induction of myogenic differentiation, both markers were detected in all groups (Figure 6A,C). Quantitative fluorescence analysis showed that MyoD and myogenin levels were significantly higher in the Hypoxia group than in the Normoxia and Hypoxia+3MA groups (Figure 6B,D). These changes were consistent with the proliferation assay results.

## 4. Discussion

In this study, we investigated the role of hypoxia-induced autophagy in regulating the proliferation and differentiation of aged skeletal muscle satellite cells (MuSCs). Our results indicate that hypoxia is associated with enhanced autophagy-related activity involving the HIF-1α/BNIP3 signaling axis, which is accompanied by improved proliferative and myogenic capacities. Pharmacological inhibition of autophagy with 3-MA markedly attenuated these effects, suggesting that autophagy contributes to the hypoxia-driven enhancement of MuSCs proliferation and differentiation.

In healthy skeletal muscle, satellite cells reside under dynamic physiological hypoxia [23,24]. With aging, microcirculation and oxygen-delivery dynamics become impaired, disrupting oxygen sensing and transcription/translation within the HIF axis of skeletal muscle satellite cells; together with dysregulation of the mitochondria–autophagy–ROS network, these perturbations mutually amplify to enforce cell-cycle inhibition, deplete stemness, and delay myogenic differentiation [25,26]. Cirillo et al. have shown that activating HIF-1α in aged skeletal muscle satellite cells can partially restore stemness and anti-atrophy phenotypes [27]. Most contemporary work modulates HIF-1α transcriptional activity via hypoxic exposure [28,29]. Consistent with these studies, we also employed hypoxia as the intervention. In the present study, CCK-8 readouts and the proliferation marker Ki67 indicated that hypoxia significantly promoted the proliferation of aged skeletal muscle satellite cells. MyoD is a lineage-determining bHLH transcription factor that drives satellite cell activation and commitment; its upregulation promotes myogenic entry and the transcriptional induction of myogenin [30]. We found that the expression levels of myogenic differentiation factors were significantly higher in the hypoxia group than in the normal group, indicating that hypoxia-induced autophagy exerts a significant promotive effect on myogenic differentiation.

Wen et al. indicate that young satellite cells maintain relatively high autophagic activity to avert entry into senescence [31], whereas autophagic flux progressively declines with age, impairing proliferation and differentiation [32]. In this study, we established an in vitro senescence model of MuSCs by serial passaging. Senescence was assessed by senescence-associated β-galactosidase (SA-β-gal) staining [33], manifested as an increased number of blue-positive cells. The proportion of SA-β-gal–positive cells was significantly higher in the old group than in the young group, indicating cellular senescence. In old MuSCs, p62 protein expression was elevated, accompanied by reciprocal changes in Beclin-1 and in the LC3B-II/LC3B-I ratio, indicating reduced autophagic activity; these findings are consistent with previous reports [26,34].

Hypoxia induces autophagy by enhancing the transcriptional activity of HIF-1α and upregulating BNIP3 expression [19,35]. Although the HIF-1α/BNIP3 axis is a recognized mechanism of hypoxia-induced autophagy, its role in aged skeletal muscle satellite cells remains insufficiently explored. Therefore, using 3% O_2_, we evaluated the regulatory effect of a hypoxic microenvironment on autophagic activity in old MuSCs. We observed marked increases in intracellular HIF-1α and BNIP3 under hypoxia group. Concordantly, Beclin-1 expression and the LC3B-II/LC3B-I ratio were higher than in the Normoxia group, whereas p62 was significantly reduced, consistent with enhanced autophagy-related activity. Upon addition of 3-methyladenine (3-MA), the expression patterns of these autophagy-related proteins were reversed. Collectively, these findings indicate that hypoxia activates autophagy in old MuSCs, consistent with engagement of the HIF-1α/BNIP3 pathway, and that 3-MA abrogates this response, consistent with an involvement of autophagy.

Using an in vitro model of aged skeletal muscle satellite cells subjected to hypoxic intervention, this study has several limitations that should be acknowledged. First, autophagy was primarily assessed based on the expression of LC3B, Beclin-1, and p62, which are widely used markers reflecting autophagosome formation and substrate turnover under steady-state conditions. Although coordinated changes in these markers are commonly interpreted as indicative of altered autophagy-related activity, such measurements do not allow a definitive distinction between enhanced autophagosome formation and impaired autophagic degradation. In addition, we did not systematically delineate the relationship between autophagy-related changes and mitochondrial functional readouts, including membrane potential, respiratory capacity, and reactive oxygen species levels. Future studies incorporating dynamic autophagic flux assays together with detailed mitochondrial analyses will be necessary to further clarify the regulation of autophagy under hypoxic conditions.

Second, this study relied on 3-MA as a pharmacological inhibitor to probe the involvement of autophagy. Although 3-MA is commonly employed to inhibit autophagosome formation through suppression of class III phosphatidylinositol 3-kinase (PI3K), it is also known to exert off-target and context-dependent effects on class I PI3K signaling pathways, which may influence cellular processes such as proliferation and survival independently of autophagy. Therefore, the effects observed following 3-MA treatment should be interpreted with caution, and 3-MA should be regarded as a supportive rather than definitive tool for mechanistic inference. More specific genetic or pharmacological approaches will be required in future studies to further substantiate the role of autophagy in hypoxia-mediated cellular responses at the mechanistic level.

Finally, the aged MuSC model in this study was generated by replicative senescence through serial passaging in vitro. While this widely used approach is practical, it does not fully recapitulate physiological aging in vivo, which is shaped by systemic factors such as hormonal regulation, immune interactions, and the stem cell niche. Moreover, senescence was primarily assessed by SA-β-gal staining, which—although commonly used—is not definitive on its own. Accordingly, the physiological relevance and robustness of our findings warrant further validation using MuSCs isolated from aged animals and complementary in vivo aging models, together with additional senescence markers (e.g., p16^INK4a^, p21^Cip1^, or Lamin B1).

In summary, hypoxia induces autophagy via the HIF-1α/BNIP3 signaling pathway, thereby promoting the proliferation and myogenic differentiation of aged MuSCs. Pharmacologic inhibition of autophagy attenuated these effects, supporting a role for autophagy in mediating the effects of hypoxia-driven enhancement of proliferation and differentiation in aged MuSCs. Using molecular, phenotypic, and functional evidence, this study proposes a conceptual hypoxia–autophagy–functional restoration framework that may inform new strategies for addressing age-related impairments in muscle regeneration.

## Data Availability

The data that substantiate this study’s conclusions are obtainable from the corresponding author upon reasonable request.

## Abbreviations

The following abbreviations are used in this manuscript:

MuSCs	skeletal muscle satellite cells
FBS	fetal bovine serum
P/S	penicillin–streptomycin
PBS	phosphate-buffered saline
SA-β-gal	senescence-associated β-galactosidase
3-MA	3-methyladenine
SDS–PAGE	SDS–polyacrylamide gel electrophoresis
TBST	Tris-buffered saline with 0.1% Tween-20
BNIP3	Bcl-2/adenovirus E1B 19 kDa-interacting protein 3
MyoD	myogenic differentiation 1
DAPI	4′,6-diamidino-2-phenylindole
WB	Western blotting
CCK-8	Cell Counting Kit-8
DMEM	Dulbecco’s modified Eagle’s medium
SEM	standard error of the mean
ANOVA	analysis of variance
Ki67	proliferation marker protein Ki-67
LC3B	microtubule-associated protein 1 light chain 3 beta
